# Some Remarks on Hernia

**Published:** 1908-11-28

**Authors:** 


### SOME REMARKS ON HERNIA.

### VI.—THE TREATMENT OF A STRANGULATED HERNIA.

A strangulated hernia demands immediate treatment. If the condition is allowed to persist unrelieved the patient will assuredly die.

The patient is at once put to bed and surrounded by hot-water bottles and warm blankets to overcome his shock. This may take half an hour. An attempt may then be made to reduce the hernia by taxis. This will be much facilitated if during the previous half-hour the foot of the bed has been raised and a warm fomentation containing tincture of opium has been applied to the swelling.

It is a debatable point how long taxis should be persisted in. The answer is that no definite limit of time can be given; each case must be judged on its own merits. A more prolonged attempt may be made, for instance, in a case in which the symptoms are not very acute than in one in which the general condition of the patient is grave. One hard and fast rule, however, may be laid down. The manipulation should always be gently performed, the pressure being brought to bear from the wrist only, and not with the whole force of the arm. If this precaution is neglected much harm may be done to the patient, the strangulated coils being still further damaged.

As a general rule one may say that it is not advisable to try taxis for more than three or four minutes. If reduction has not occurred by then operation should not be delayed. In any case the reduction of a strangulated hernia by taxis should only be regarded as a temporary measure, except in extremely aged patients in whom an operation is undesirable; for the patient is left with a hernia which is quite likely to become strangulated again on a subsequent occasion, and it is therefore better for him to have a radical cure performed at once than to postpone it indefinitely. Then, again, taxis which is apparently successful is not always really so; and a percentage of cases, though admittedly a small one, die even though reduction seems to have been successfully accomplished; or, at any rate, the symptoms may not be relieved. This may be due to a variety of contingencies. The most common is that the hernia is returned to the abdomen, sac and all; and, the neck of the sac being the constricting agent, the symptoms necessarily continue. Or gangrene may supervene in the intestine which has been down in the hernia after it has been reduced. Or, again, the gut may have been so much damaged that, even though reduced, it has lost its tone and refuses to perform its natural functions.

In any of these cases the symptoms of acute intestinal obstruction continue, and the abdomen must then be opened to discover and, if possible, to deal with the cause.

Taxis should not be attempted at all in any of the following circumstances: (1) In a hernia which has been irreducible for a long time; (2) in one which has been strangulated for many hours; or (3) in one in which the symptoms are exceedingly acute.

If, therefore, taxis is contra-indicated or has been tried without success, the patient should be anæsthetised as soon as possible and operative treatment undertaken. The main points of the operation are the same whether it be undertaken for an inguinal or femoral hernia, the principal difference being that in the one the constricting agent is the neck of the sac, whereas in the other it is Gimbernat's ligament.

An incision is made over the swelling, oblique in the case of an inguinal, and vertical in the case of a femoral hernia. When the sac is reached, it is often found to be shiny, thickened and purplish in colour. In fact, it may very closely resemble strangulated intestine. The change in colour is due to the fact that it contains blood-stained fluid. On incising the sac, the fluid escapes and the intestine comes into view. The next thing is to release the constriction. The conventional way of doing this is to pass a Keye's broad-bladed director between the intestine and the neck of the sac, and then to divide the neck with a hernia knife. This is a scalpel with a blunt, rounded point and a short, cutting edge about half an inch from the end of the blade. It is so constructed that when it is introduced no damage will be done to the structures beyond. The constriction is then divided by a series of snicks, until the intestine can be pulled down and examined. There are two points to observe about the strangulated gut: one is its general condition and the probability of its being able to recover; and the second is the state of affairs at the point of constriction. The intestine may be plum-coloured, or even black, and still it may recover. A point of greater value to notice is whether its surface is still shiny or not. If it is, it may be safely returned to the abdomen. Before doing this, it should be pulled down and thoroughly examined at the place where it was constricted. It will often be found to be ulcerated at this site. In this case the ulcers should be buried with one or two rows of Lembert's sutures. If there is no doubt whatever as to its viability it may be returned to the abdomen, and a radical cure may be performed in the ordinary way. In the case of an inguinal hernia the only difficulty of the operation consists in keeping the coils from wrapping round the director and being injured by the hernia knife while the constriction is being divided. But in a femoral hernia there is an added risk. In a small percentage of cases the obturator artery has an abnormal origin from the deep epigastric and runs downwards behind Gimbernat's ligament. Thus it may be cut by the hernia knife, for in a femoral hernia the constricting agent is the sharp edge of Gimbernat's ligament, and this is divided by turning the edge of the knife inwards and incising the ligament. If an abnormal obturator artery is cut, it retracts out of the field of operation. It is an artery of sufficient size to give rise to serious hæmorrhage, and in this event the deep epigastric artery must be exposed and ligatured. M. Doyen, of Paris, advocates releasing the constriction not by cutting, but by inserting a pair of scissors with the blades closed, and stretching the ring by opening the blades subsequently. This method avoids both the dangers mentioned. The operative steps to be taken, when it is unlikely that the gut will survive, will be discussed in a succeeding article.

